# Supplementary material for: Silk Fibroin-Induced Gadolinium-Functionalized Gold Nanoparticles for MR/CT Dual-Modal Imaging-Guided Photothermal Therapy
Source: J Funct Biomater. 2022 Jun 22;13(3):87. doi: 10.3390/jfb13030087 (PMC9326592; doi:10.3390/jfb13030087)
Supplement: Supplementary file 1 [file jfb-13-00087-s001.zip › jfb-1748238-supplementary.pdf]

Silk fibroin-induced gadolinium-functionalized gold nanoparticles for MR/CT dual-modal imaging-guided photothermal therapy

Chuanxue Yang[1,#], Tianxiao Mei[1,2,#], Qingge Fu[3,#], Yifan Zhang[1], Yang Liu[1], Ran Cui[1,2], Gang Li[4], Yibin Wang[4], Jianguo Huang[1], Junqiang Jia[5,*], Bo Chen[1,2,*], Yihui Hu[1,*]

[1] *Institute for Regenerative Medicine, Jian Hospital & Shanghai East Hospital, School of Life Sciences and Technology & School of Medicine, Tongji University, Shanghai 200092, China*

[2] *Department of Hepatopancreatobiliary Surgery, Jian Hospital & Shanghai East Hospital, Jian, Jiangxi 343000 China*

[3] *Department of Orthopedic trauma, Changhai Hospital, Naval Medical University, Shanghai 200433, China*

[4] *Department of Radiology, Shanghai East Hospital, School of Medicine, Tongji University, Shanghai 200092 China*

[5]*School of Grain Science and Technology, Jiangsu University of Science and Technology, Zhenjiang, Jiangsu 212100, China*

Keywords: silk fibroin; AuNPs; biomimetic synthesis; MR/CT imaging; PTT

#: equally contributed
* Corresponding author; Junqiang Jia, Bo Chen, Yihui Hu
Email: jiajq@just.edu.cn; chenbo7349@sohu.com; yihuihu2020@163.com

Supporting methods

Calculation of photothermal conversion efficiency

The photothermal conversion of Gd:AuNPs@SF was evaluated by the temperature change of aqueous dispersion (250 μg/mL) as a function of time under 808 nm laser irradiation at a power density of 3.0 W/cm^2 until the solution reached a steady temperature. The temperature change of the aqueous solution was recorded by the infrared imager. The η value was calculated as follows:

$$\eta = \frac{hS(T_{max} - T_{surr}) - Q_{dis}}{I(1 - 10^{-A_{808}})} \quad (1)$$

Here, h is heat transfer coefficient, S is the surface area of the container, T_{max} is the equilibrium temperature, T_{surr} is the ambient temperature of the surroundings, Q_{dis} expresses the heat dissipation from the light absorbed by the quartz sample cell, I is incident laser power (3.0 W/cm^2), A_{808} is the absorbance of Gd:Au@SF NPs at 808 nm (0.375). hS was calculated as followed:

$$hS = \frac{\Sigma_i m_i c_{p,i}}{\tau_s} \approx \frac{cm}{\tau_s} \quad (2)$$

The m and c are the weight and specific heat capacity of solvent (water), respectively, τ_s is the heat dissipation time constant. Whereas, τ_s is calculated by plotting a linear data of cooling period with the negative logarithm using the following equation:

$$t = -\tau_s In \frac{T - T_{surr}}{T_{max} - T_{surr}} = -\tau_s In\theta \quad (3)$$

Q_{dis} represents the emitted by the solvent absorbing the 808 nm laser, measured by a control experiment of water, calculated using the following equation:

$$Q_{dis} = cm\Delta T_{H_2O} \quad (4)$$

Supporting figures and tables

Table S1. Data for photothermal conversion efficiency.

	τ_s (s)	hS (W/°C)	T_{max}-T_{surr} (°C)	Q_{dis} (W)	I (W/cm^2)	A_{808}	η
**Gd:AuNPs@SF**	205.6	0.020428	19.4	0.021	3	0.375	21.63 %

Table S2. Summarized methods for synthesizing nanoparticles involving gold and SF.

Nanoparticles	Synthetic Method	Advantages	Applications	Ref
**AuNPs**	Turkevich synthesis	Dispersity and size reproducibility	Large-scale production	[1]
**AuNPs**	Brust synthesis	High thermal stability	NA	[2]
**AuNPs**	Greener synthesis	Facile and eco-friendly method	NA	[3]
**BSA-Au NCs**	Biomineralization	“Green” method, highly fluorescent rate	Provided protocol to proteins and noble metals	[4]
**AuPt@SF NPs**	Biomineralization	Responsive to tumor microenvironmental	Tumor theranostics.	[5]
**AuNPs/SF**	Self-Assembly	High NIR absorption and photothermal conversion efficiency	Tumor photothermal therapy	[6]
**SF@MnO_2/ICG/DOX nanocomplex**	Biomineralization	Multifunction complex	Tri-modal imaging-assisted cancer therapy	[7]
**ICG-SFNPs**	Desolvation (acetone)	Low energy consumption	Tumor imaging and therapeutic	[8]
**ACC-DOX-SF NPs**	Gas diffusion Biomineralization	TME-responsive	Cancer chemotherapy	[9]
**Gd:AuNPs@SF**	Biomimetic synthesis	Multifunctional theranostic nanoplatform	MR/CT dual-modal imaging-guided photothermal therapy	This work

Table S3. The quantitative analysis of Gd and Au in main organs and tumors by ICP-OES.

Sample	Au (mg/L)				Gd (mg/L)			
	Number 1	Number 2	Number 3	mean	Number 1	Number 2	Number 3	mean
Heart	0.217	0.263	0.292	0.257	0.002	0.003	0.001	0.002
Liver	0.818	0.826	0.847	0.83	0.274	0.278	0.278	0.277
Spleen	0.887	1.079	1.175	1.047	0.021	0.019	0.02	0.02
Lung	0.687	0.792	0.839	0.773	0.773	0.019	0.018	0.019
Kidney	0.205	0.247	0.272	0.241	0.004	0.006	0.004	0.005
Tumor	0.273	0.325	0.359	0.319	0.001	0.001	0.002	0.001

Supplementary Figure S1

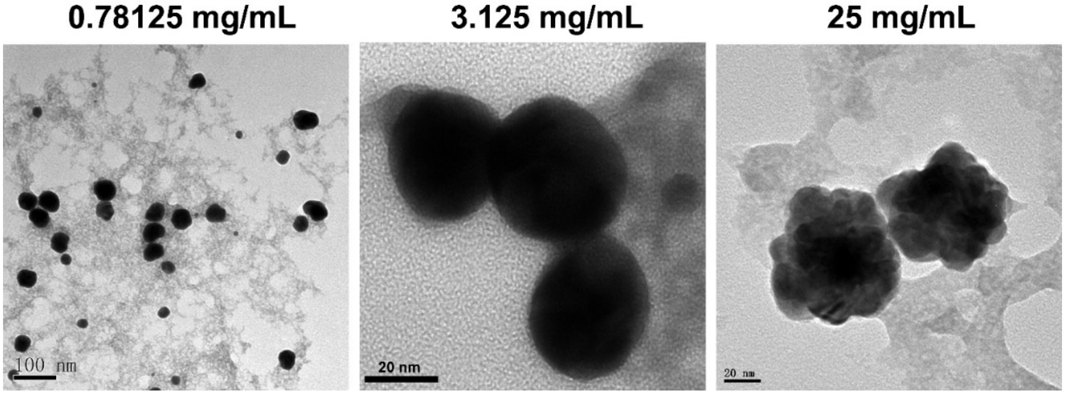

TEM imaging of Gd:AuNPs@SF with different SF concentration (0.78125, 3.125, 25 mg/mL).

Supplementary Figure S2

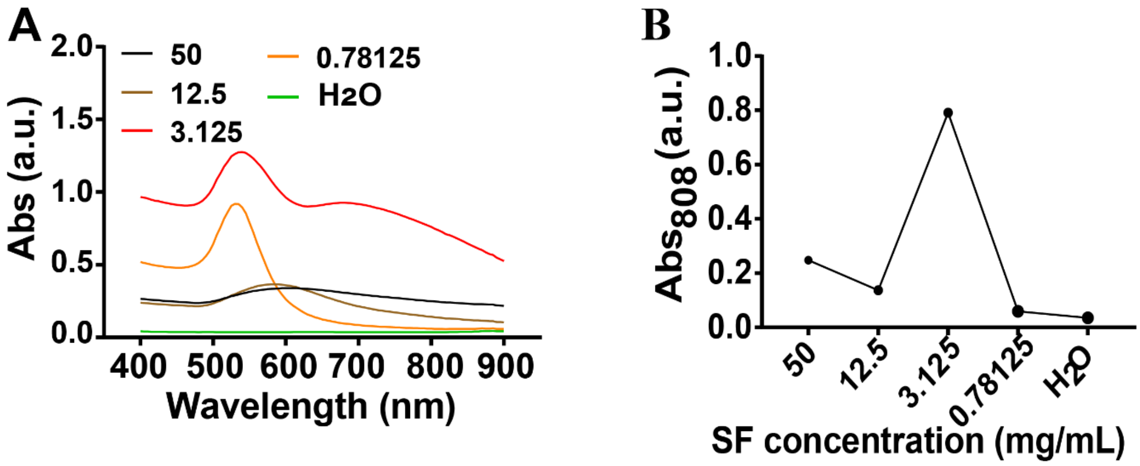

UV-vis spectra of Gd:AuNPs@SF synthesized with different SF concentrations (0.78125, 3.125, 12.5, 50 mg/mL). (**A**) UV-vis spectra of Gd:AuNPs@SF with a series of SF concentrations, and (**B**) UV-vis absorption of corresponding Gd:AuNPs@SF at 808 nm.

Supplementary Figure S3

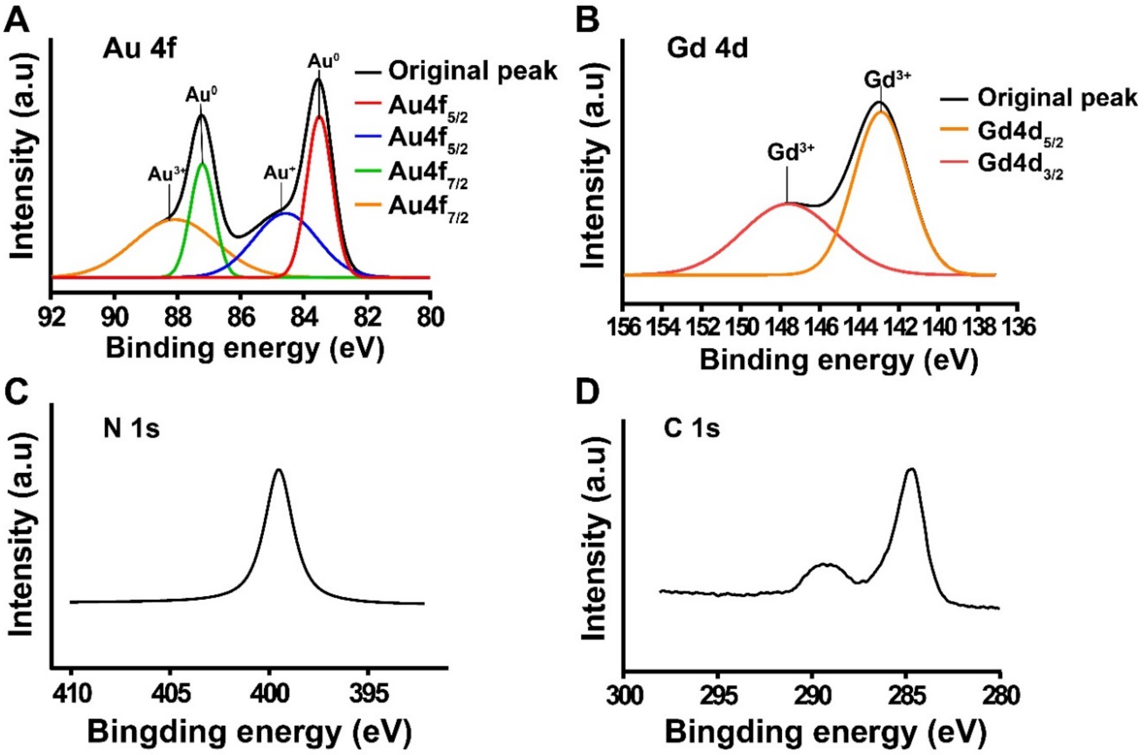

Core level XPS spectra of Au (4f), Gd (4d), N (1s), C (1s) in Gd:AuNPs@SF.

Supplementary Figure S4

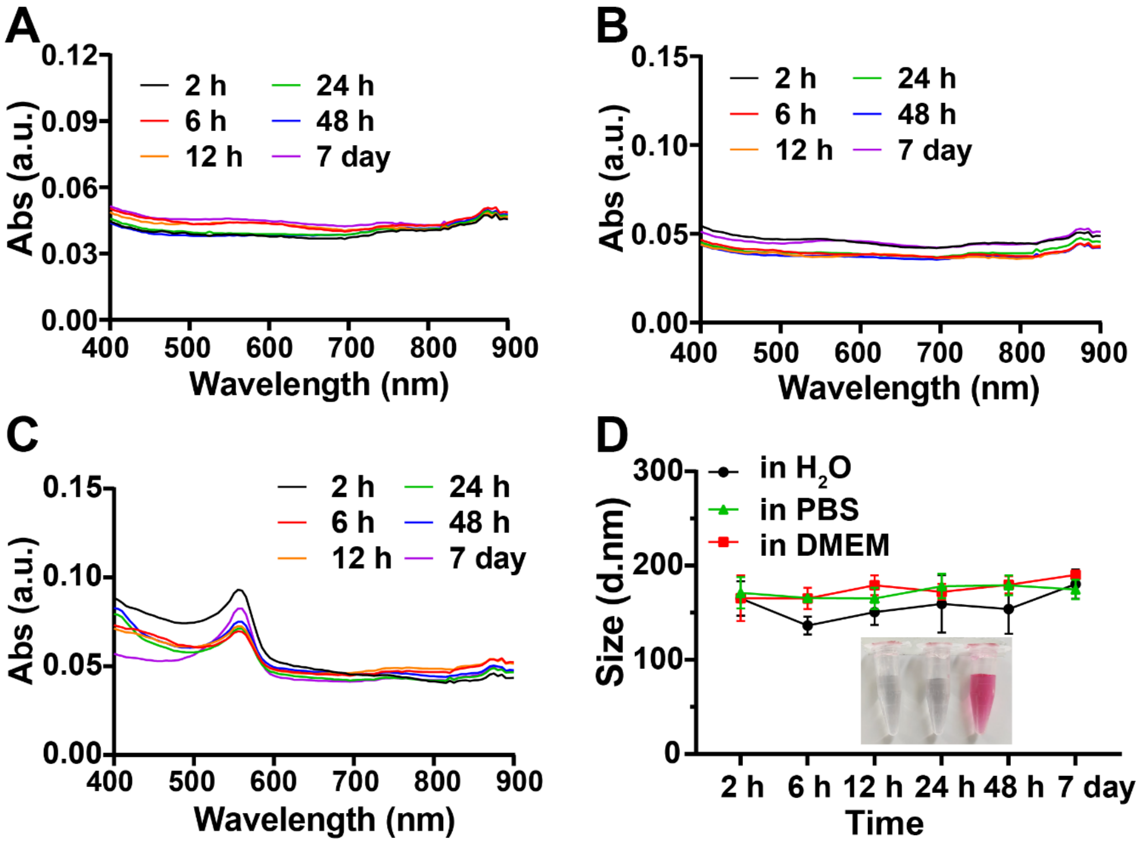

Stability analysis of Gd:AuNPs@SF. UV-vis spectra of Gd:AuNPs@SF dispersed in water (**A**), PBS (**B**), and DMEM (**C**), and the corresponding hydrodynamic diameter (**D**). The inset is a 7 days digital photo.

Supplementary Figure S5

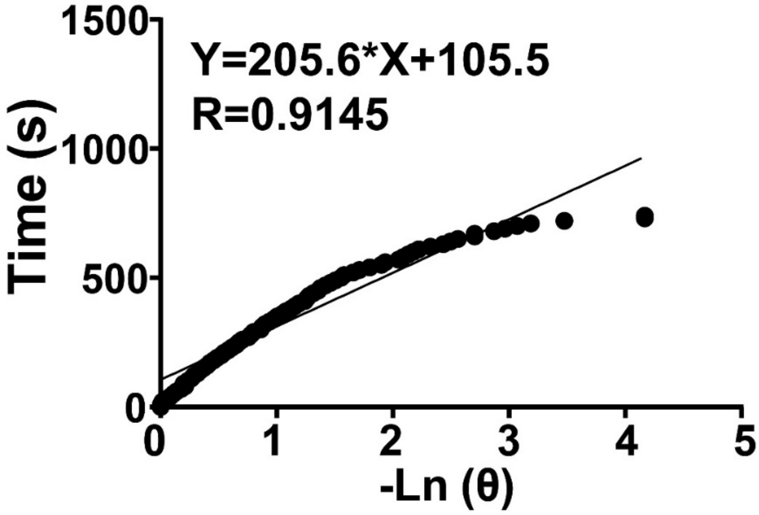

Calculation of the heat transfer constant of Gd:AuNPs@SF after irradiation by 808 nm laser.

Supplementary Figure S6

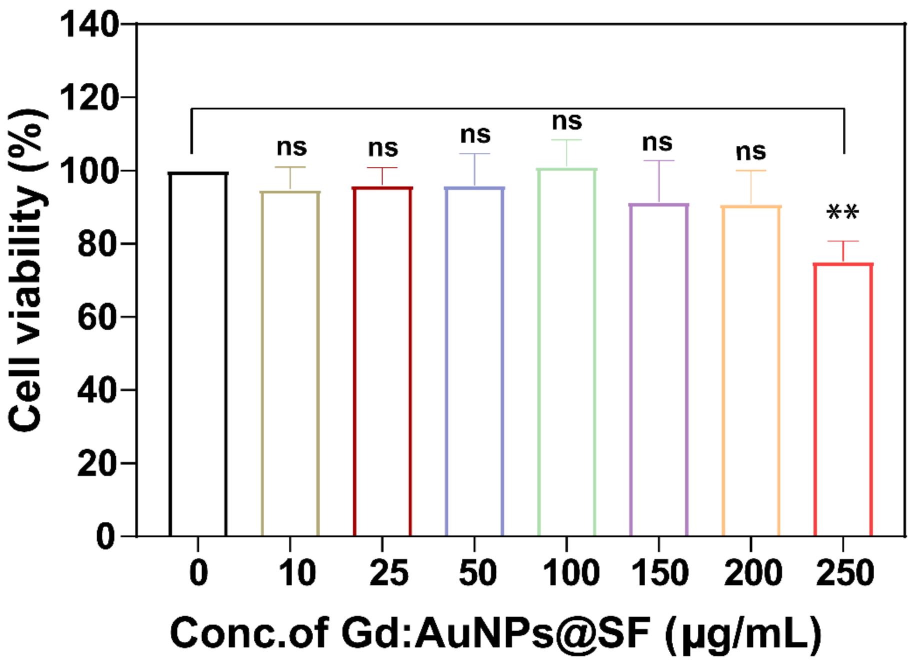

Viability of HUVEC cell after being incubated with Gd:AuNPs@SF at different concentrations for 24 h. (ns P>0.05;**P < 0.01)

Supplementary Figure S7

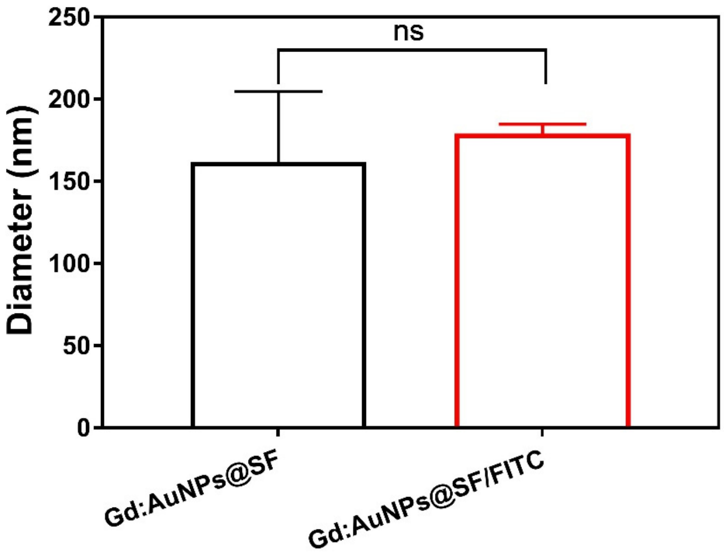

The average particle size of Gd:AuNPs@SF and Gd:AuNPs@SF/FITC. (ns P >0.05;).

Supplementary Figure S8

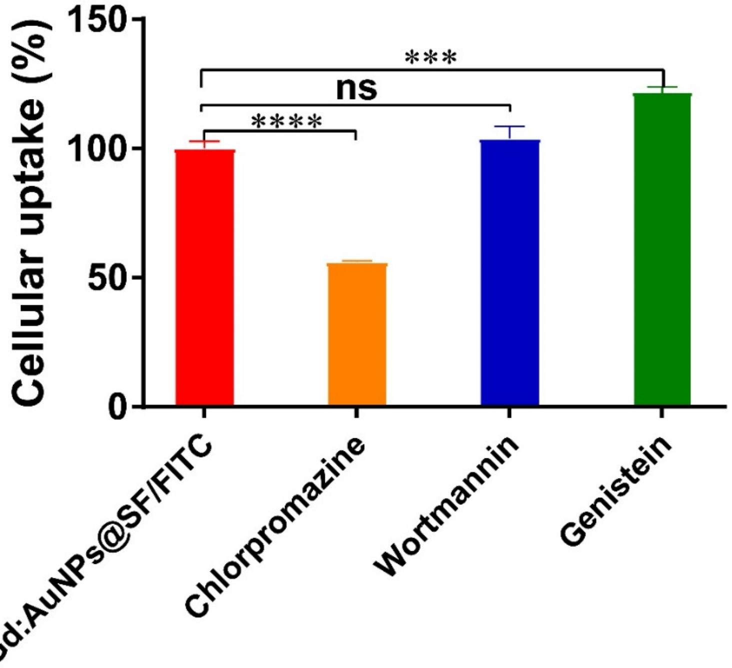

Flow cytometry assay of FITC contents inside Pan02 cells treated with Gd:AuNPs@SF/FITC in the absence or presence of chlorpromazine (10 μg/mL), genistein (200 μg/mL), or wortmannin (100 ng/mL), respectively. (ns $P > 0.05$; ***$P < 0.001$; ****$P < 0.0001$).

Supplementary Figure S9

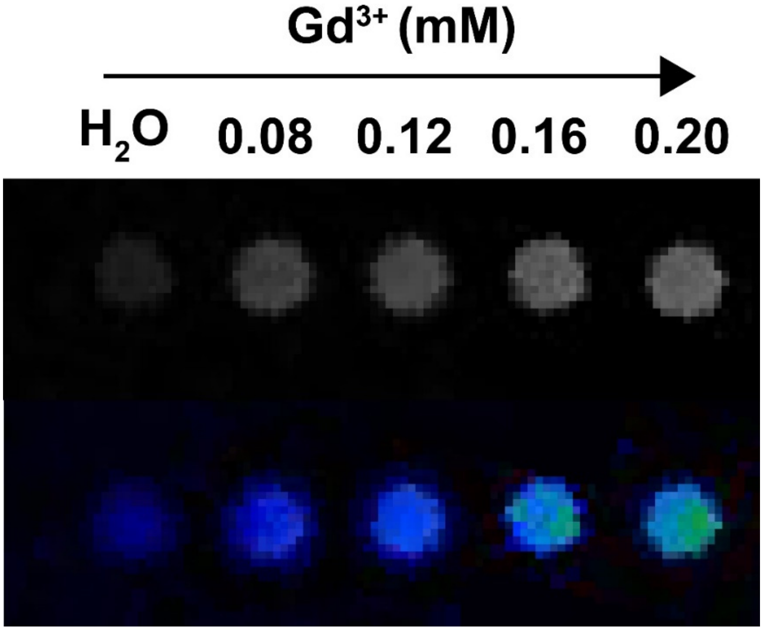

T_1-weighted and false-color-mapped of different Gd^{3+} concentrations (0-0.20 mM) of Gd-DTPA in water.

Supplementary Figure S10

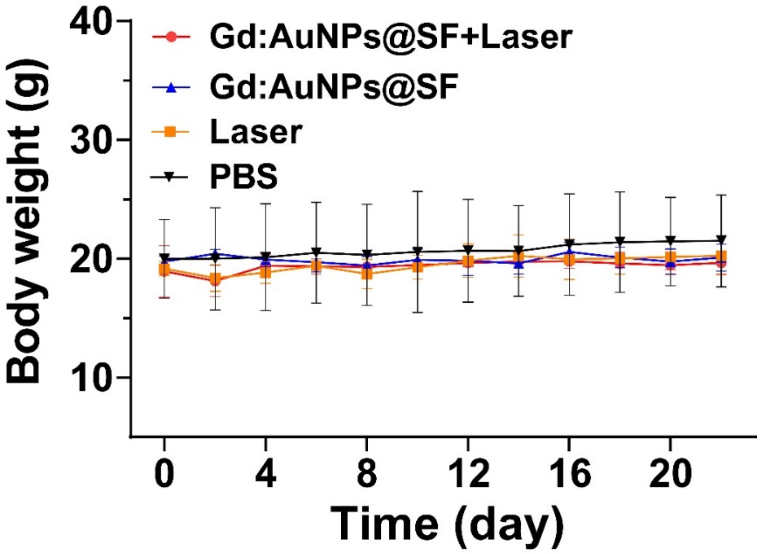

The bodyweight of tumor-bearing mice after being treated by Gd:AuNPs@SF + laser, Gd:AuNPs@SF, laser, PBS for 22 days.

Supplementary Figure S11

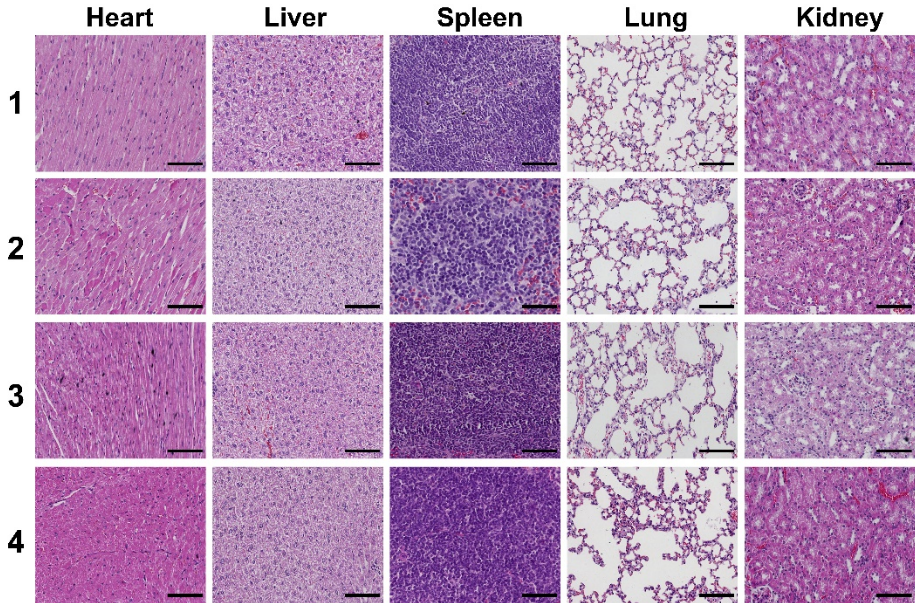

H&E staining images of the main organs (heart, liver, spleen, lung, and kidney) were collected from the mice after 22 days of photothermal treatment (scale bare: 100 µm). 1: Gd:AuNPs@SF + Laser, 2: Gd:AuNPs@SF, 3: Laser, 4: PBS.

References

1. Schulz F, Homolka T, Bastús NG, Puntes V, Weller H, Vossmeyer T. Little adjustments significantly improve the Turkevich synthesis of gold nanoparticles. *Langmuir*, **2014**, *30*, 10779-10784.
2. Brust M, Walker M, Bethell D, Schiffrin DJ, Whyman R. Synthesis of thiol-derivatised gold nanoparticles in a two-phase Liquid–Liquid system. *J Chem Soc, Chem Commun*, **1994**, *7*. 801-802.
3. Kharissova OV, Dias HV, Kharisov BI, Pérez BO, Pérez VM. The greener synthesis of nanoparticles. *Trends Biotechnol*, **2013**, *31*, 240-248.
4. Xie J, Zheng Y, Ying JY. Protein-directed synthesis of highly fluorescent gold nanoclusters. *J Am Chem Soc*, **2009**, *131*, 888-889.
5. Yang R, Fu S, Li R, Zhang L, Xu Z, Cao Y, et al. Facile engineering of silk fibroin capped AuPt bimetallic nanozyme responsive to tumor microenvironmental factors for enhanced nanocatalytic therapy. *Theranostics*, **2021**, *11*, 107-116.
6. Wang J, Zhang Y, Jin N, Mao C, Yang M. Protein-Induced Gold Nanoparticle Assembly for Improving the Photothermal Effect in Cancer Therapy. *ACS Appl Mater Interfaces*, **2019**, *11,* 11136-11143.
7. Yang R, Hou M, Gao Y, Lu S, Zhang L, Xu Z, et al. Biomineralization-inspired Crystallization of Manganese Oxide on Silk Fibroin Nanoparticles for in vivo MR/fluorescence Imaging-assisted Tri-modal Therapy of Cancer. *Theranostics*, **2019**, *9*, 6314-6333.
8. Xu HL, ZhuGe DL, Chen PP, Tong MQ, Lin MT, Jiang X, et al. Silk fibroin nanoparticles dyeing indocyanine green for imaging-guided photo-thermal therapy of glioblastoma. *Drug Deliv*, **2018**, *25*, 364-375.
9. Tan M, Liu W, Liu F, Zhang W, Gao H, Cheng J, et al. Silk Fibroin-Coated Nanoagents for Acidic Lysosome Targeting by a Functional Preservation Strategy in Cancer Chemotherapy. *Theranostics*, **2019**, *9*, 961-973.
